# Genetic diversity of *Plasmodium vivax* in clinical isolates from Bangladesh

**DOI:** 10.1186/s12936-015-0790-4

**Published:** 2015-07-11

**Authors:** Mohammad Golam Kibria, Rubayet Elahi, Abu Naser Mohon, Wasif A Khan, Rashidul Haque, Mohammad Shafiul Alam

**Affiliations:** International Centre for Diarrhoeal Disease Research Bangladesh (icddr,b), Dhaka, 1212 Bangladesh; Department of Biochemistry, Virginia Tech, Blacksburg, VA 24061 USA; Department of Microbiology and Infectious Disease, Cumming School of Medicine, University of Calgary, Calgary, AB T2N1N4 Canada

**Keywords:** *Plasmodium vivax*, Genetic diversity, *Pvcsp*. *Pvmsp 1*, *Pvmsp 3α*, Bangladesh

## Abstract

**Background:**

*Plasmodium vivax* is the second most prevalent human malaria parasite in Bangladesh; however, there are no data of its genetic diversity. Several molecular markers are available where *Pvcsp*, *Pvmsp 1* and *Pvmsp 3α* are most commonly used for *P. vivax* genotyping studies. The aim of the study was to investigate the population structure of *P. vivax* in Bangladesh.

**Methods:**

A total of 102 *P. vivax*-positive blood samples were collected from different malaria-endemic areas in Bangladesh and subsequently analysed for those three genotyping markers. Nested PCR was performed for diagnosis and genotyping analysis followed by PCR–RFLP to detect genetic diversity using *Pvcsp*, *Pvmsp 1* and *Pvmsp 3α* markers.

**Results:**

Analysis of *Pvcsp* showed that the VK210 repeat type was highly prevalent (64.7%, 66/102) compared to VK247 (35.3%, 36/102), although the prevalence of VK247 was higher than other Southeast Asian countries. Analysis of these three genes revealed a diverse, circulating population of *P. vivax* where a total of ten, 56 and 35 distinct genotypes were detected for *Pvcsp*, *Pvmsp 1* and *Pvmsp 3α*, respectively.

**Conclusion:**

This genotyping observation of *P. vivax* is the first report from Bangladesh and will provide valuable information for establishing the genotyping methods and circulating genetic variants of these three markers available in Bangladesh.

## Background

Approximately 3.2 billion people were at risk of malaria globally in 2013 [1]. Among five *Plasmodium* species causing malaria in humans, *Plasmodium falciparum* is the most deadly and predominant, followed by *Plasmodium vivax*, which is less virulent but has a wide geographical distribution. About 8% of estimated cases globally are due to *P. vivax*, but its incidence outside the African continent is in similar proportion to *P. falciparum* [1]. Although once thought benign, *P. vivax* has recently been found to be associated with severe anaemia, respiratory distress, malnutrition [2], and recurrent haemolysis [3]. It has been reported from clinical findings from Thailand and India that vivax malaria during pregnancy causes maternal anaemia and a significant reduction in mean birth weight [4, 5].

Drug resistance is a growing problem. Resistance to sulfadoxine-pyrimethamine (SP) and chloroquine by *P. falciparum* has led to an increase in morbidity and mortality [6]. Worryingly, SP and chloroquine resistance have also been reported for *P. vivax* recently [6–8], which calls for measures to restrict the spread of drug resistance. One of the important tools to monitor drug resistance is to study the molecular markers in malaria parasites involved in consecutive transmission [9].

Examining the genetic diversity and population structure of *P. vivax* parasites provides insights into the transmission dynamics of vivax malaria, which are important to help support and monitor malaria control measures, including the design and evaluation of new drugs and vaccines [10, 11]. Various large-scale studies have been conducted for *P. falciparum* and the presence and dynamics of different single or multiple polymorphic genes encoding different antigens have been investigated [12–14]. In recent studies, well-characterized polymorphic antigenic regions in both pre-erythrocytic and erythrocytic genes have been widely used to analyse genetic diversity patterns in *P. vivax* populations [15–17]. For molecular genotyping studies of *P. vivax*, three polymorphic single copy genes have been in common use: *Pvcsp* coding for the circumsporozoite protein, which is responsible for binding of sporozoite to liver cells and contains two types of repeat elements (either VK210 or VK247) [15, 18, 19]; *Pvmsp 1* (coding for the merozoite surface protein 1), which is involved in the parasite’s invasion to red blood cells and contains 13 inter-allele conserved and highly variable blocks, where variable blocks are: block 2 (F1 region), 6–8 (F2 region) and 10 (F3 region) [18]; and, *Pvmsp 3α*, which initiates antibody-dependent, cell-mediated inhibition during repeated malaria infection by triggering the binding of antibody to monocytes and consists of an alanine-rich central domain [20].

While *P. vivax* is the second most malaria-causing parasite in Bangladesh [21], it receives relatively little attention [22]. There is no information available on the circulating strains of *P. vivax* across endemic areas of Bangladesh. The primary objective of this study was to document the genetic diversity of *P. vivax* by three well-established markers (*Pvcsp, Pvmsp 1* and *Pvmsp 3α*) in some selected endemic areas of Bangladesh.

## Methods

### Study sample

**Figure 1:**
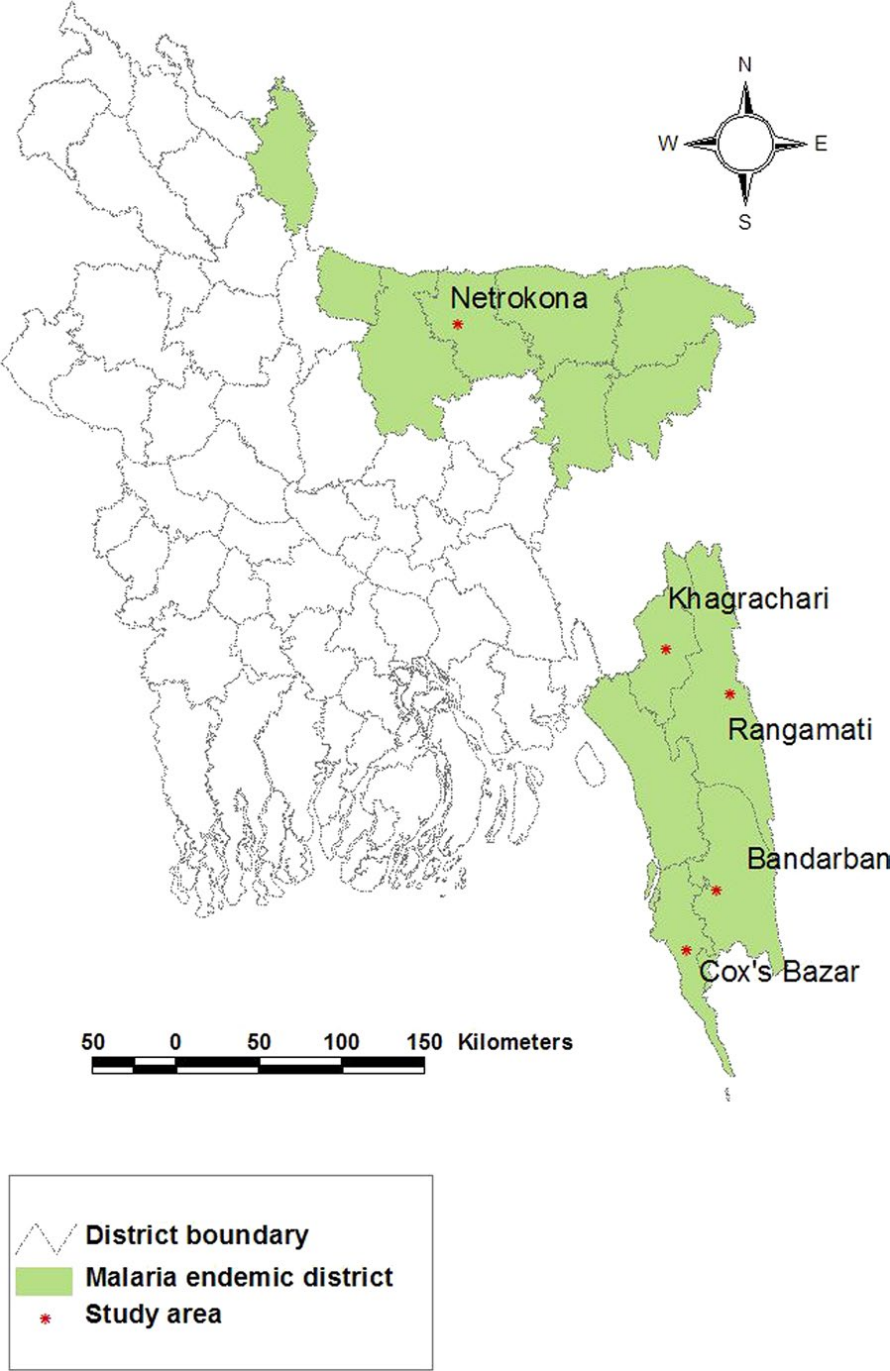
Geographical map of the study areas.

A total of 102 *P. vivax* mono-infected blood samples (day 0) from five malaria-endemic districts in Bangladesh: Bandarban (9), Cox’s Bazar (73), Khagrachari (15), Rangamati (1), and Netrokona (4), were considered for this study. The age range of patients was 2–50 years, with median age of 25.5 years. All the samples were collected from patients with febrile illness at different *upazila* (sub-district) health complexes (UHC) of the aforementioned districts (Figure 1) from May 2009 to April 2014, but no samples were collected in 2011 (total 35, 39, four, four, and 20 samples were collected in the years 2009, 2010, 2012, 2013, and 2014, respectively). These samples were referred to microscopy for malaria diagnosis. No follow-up data were collected from any of the patients. All samples were positive in microscopy and nested PCR [23] and/or real-time PCR for *P. vivax* mono-infection [22]. Most of the samples were used for different studies reported elsewhere [22, 24, 25] and the studies were approved by the Research Review Committee and Ethical Review Committee of the International Centre for Diarrhoeal Disease Research Bangladesh (icddr,b). All the patients consented to further use of blood samples.

### Genotyping PCR

Positive samples were analysed using nested PCR for *Pvcsp*, *Pvmsp 1* (containing variable blocks 2, 6–8 and 10, designated as F1, F2 and F3, respectively) and *Pvmsp 3α* markers as described previously [9, 20]. All amplification reactions were carried out with some modifications. Briefly, for the nested round of the PCR, reaction volume was 50 µL, where 2.5 µL of 50 times diluted initial PCR product was used as template. The PCR products were analysed by ethidium bromide stained 1.5% (for *Pvcsp* and *Pvmsp 1*) and 0.8% (for *Pvmsp 3α*) agarose gel electrophoresis.

### RFLP analysis of *Pvcsp, Pvmsp 1* and *Pvmsp 3α*

Restriction enzymes *Alu I*, *Bst NI*, *Scr FI*, *Bbs I,* and *MboII* for *Pvcsp*; *Alu I* and *Mnl I* for F2 region of *Pvmsp 1,* and *Hha I* and *Alu I* for *Pvmsp 3α* were used, as described previously for restriction fragment length polymorphism analysis of the PCR products (PCR–RFLP) [9, 20]. In brief, 10 μL of the amplified PCR product were digested individually with the restriction enzyme in 20 µL reaction volume at 37°C for 3 h. All the restriction enzymes were obtained from New England Biolabs Inc, USA. Electrophoresis was performed on ethidium bromide stained 1.5% agarose gel in TBE buffer, and was visualized under UV illumination.

### Allele detection

The repeat types of *Pvcsp* were classified based on restriction digestion [9]. Analysis of number of genotypes and size polymorphisms in *Pvcsp*, *Pvmsp 1* F1, F2, F3, and *Pvmsp 3α* was done by grouping the band sizes of electrophoresis, differing by 25 bp. Thereafter, each 25-bp interval was defined as a distinct genotype [14, 26]. All the data were computed and analysed in Microsoft Excel 2007 (Microsoft Corp, USA) and Chi square (χ^2^) test was performed to compare genetic diversity of markers with study districts using SPSS 19.0 (IBM Corp, USA). A *p* value of ≤0.05 was considered significant.

### Heterozygosity (H_E_) and mean multiplicity of infection (MOI)

The expected heterozygosity (H_E_) was calculated by using the formula ${\text{H}}_{\text{E}} = \left[ {{\text{n}}/\left( {{\text{n}} - 1} \right)} \right] \, [( 1- \varSigma {\text{P}}_{\text{i}}^{ 2} )]$, where n = sample size, P_i_ = allele frequency. The theoretical probability of infection by two parasites with the same allele was calculated as ${\text{P}} = \varSigma {\text{P}}_{\text{i}}^{ 2}$ [27]. The combined probability was calculated by multiplying the probabilities P for all marker genes, which indicates that two independent clones share the same genotype for all marker genes, assuming that all the loci sort individually from each other. The mean multiplicity of infection (MOI) was calculated by dividing the total number of clones by the number of PCR-positive samples for each marker gene.

## Results

### Allelic diversity of *Pvcsp*

**Figure 2:**
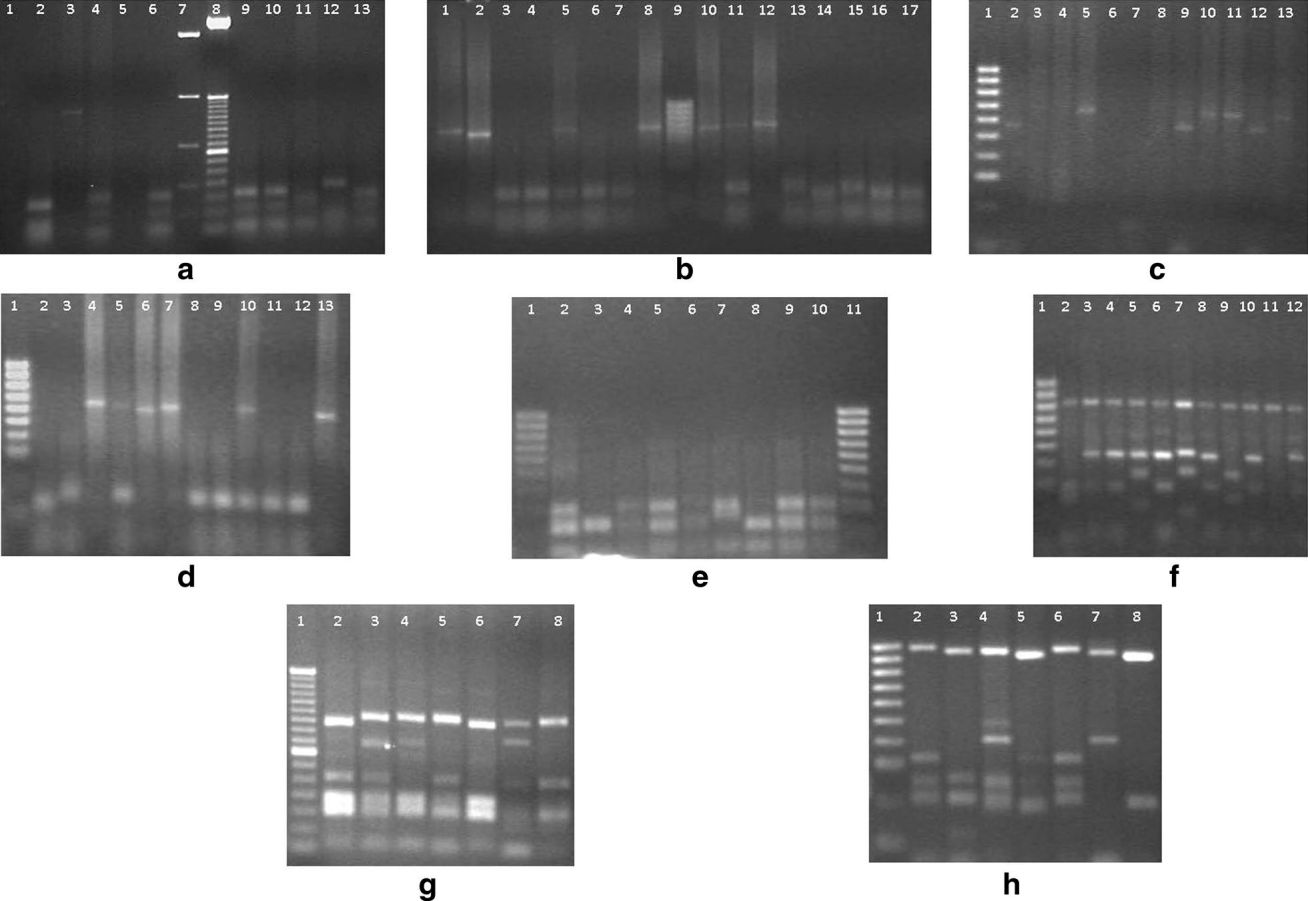
RFLP gel pictures of three markers. **a** *Alu I* digestion of *Pvcsp* PCR products for VK210 type, digested (*lane* *2, 4, 6, 9–13*), undigested (*lane 3*), marker [*lane 7, 8* (100 bp)]. **b** *Bst NI* digestion for VK247 type, digested (*lane 3–7, 11, 13–17*), undigested (*lane 1, 2, 8, 10, 12*). **c** Digestion of *Pvcsp* PCR product with *Bbs I* for presence of post repeat sequence, digested (*lane 2, 4, 6–9, 12*), undigested (*lane 3, 5, 10, 11, 13*), 100 bp marker (*lane 1*). **d** *Scr FI* digestion of *Pvcsp* product for pre-repeat site, digested (*lane 1, 2, 5, 8, 9, 11, 12*), undigested (*lane 4, 6, 7, 10, 13*), 100-bp marker (*lane 1*). **e**, **f** Restriction digestion pattern of *Pvmsp*-*1* F-2 PCR product using *Mnl I* and *Alu I,* respectively, 100-bp marker was used in both gel. **g**, **h** Digestion pattern of *Pvmsp 3α* PCR product using *Alu I* and *Hha I,* respectively. Presence of a common 1,000-bp fragment observed for *Hha I* digestion. 50-bp and 100-bp markers (*lane 1* of both gel) were used in *Alu I* and *Hha I* digestions, respectively.

**Figure 3:**
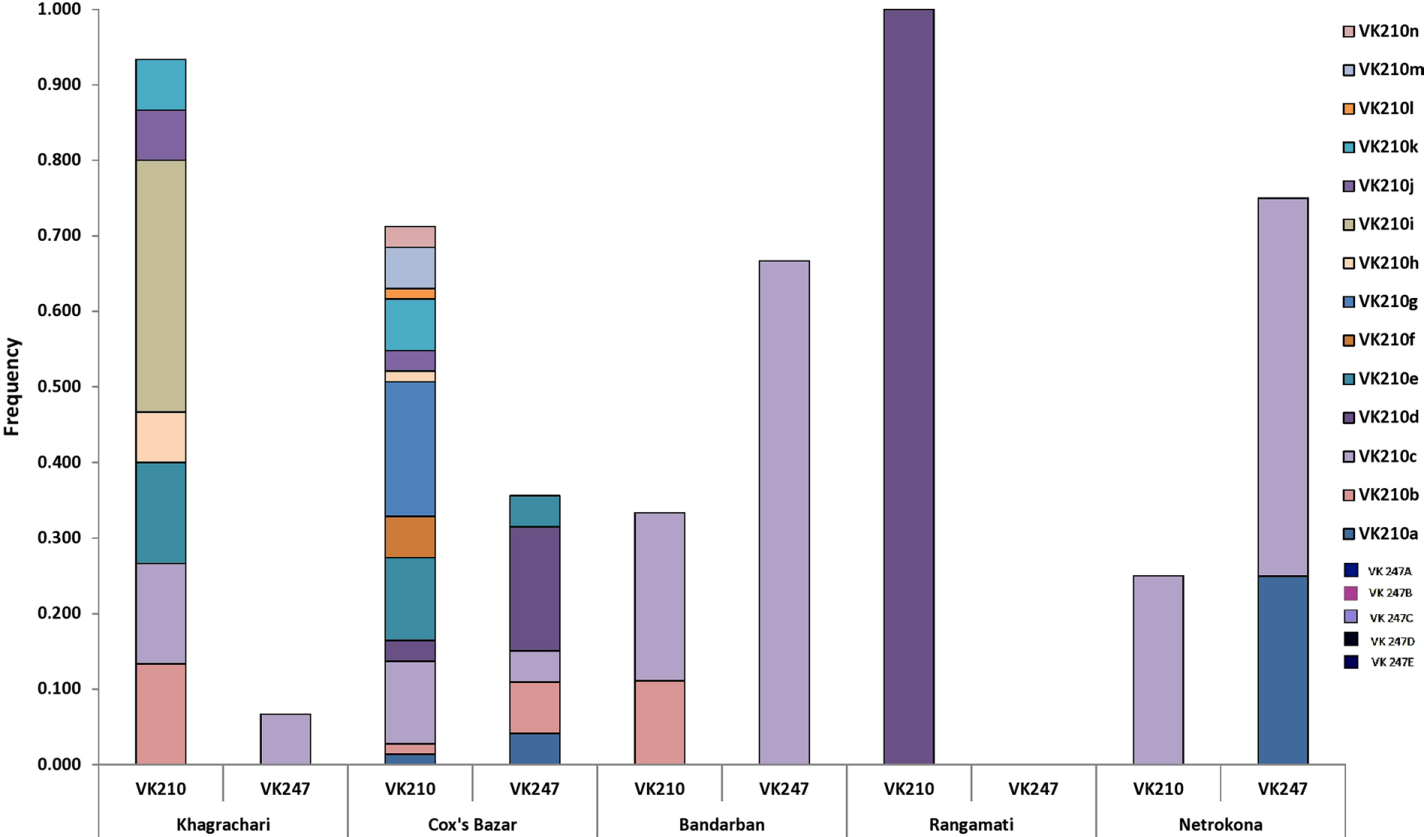
Distribution of *Pvcsp* repeat types and presence of pre- and post-repeats in Bandarban, Cox’s Bazar, Khagrachari, Rangamati, and Netrokona.

PCR–RFLP analysis of *Pvcsp* by *AluI* and *BstI* of 102 isolates revealed the presence of both VK210 (66/64.7%) and VK247 (36/35.3%) repeat types (Figure 2). Digestion products were less than 150 bp for both digestion. No isolate harboured both VK210 and VK247 repeat type infection. For *Pvcsp*, ten different allelic variants were detected, equally (five from each) representing the VK210 and VK247 repeat types. Each genotype was labelled alphabetically from A to E (650–775 bp) for both repeat types. Variant B was found abundant (0.250) for VK210, whereas for VK247, the highest frequency (0.125), was found with variant C and D. To increase the genotyping resolution of *Pvcsp*, the presence and absence of pre- and post-repeat region of VK210 (recognized by *Scr FI* and *Bbs I*, respectively) and pre-repeat region of VK247 (recognized by *MboII*) were analysed by RFLP. A total of 14 different allelic types was found for VK210 repeat type after RFLP (Table 1). Geographical distributions of both repeat types are provided in Figure 3. These allelic variants were found from 109 bands upon PCR; the highest frequency, 0.127, was found for both VK210c and VK210g (Table 1). Among these two variants, VK210c was found almost equally distributed in the study areas while VK210g was found only in Khagrachari and Cox’s Bazar (Figure 3).

**Table 1:** Allelic variant frequency of *Pvcsp* by size, repeat types and insertion of pre- and post-repeats

Allele	Size	Pre-repeat	Post-repeat	n	Frequency
VK210a	A (650–675 bp)	Yes	Yes	1	0.010
VK210b	A (650–675 bp)	Yes	No	4	0.039
VK210c	A (650–675 bp)	No	No	13	0.127
VK210d	B (675–700 bp)	Yes	Yes	3	0.029
VK210e	B (675–700 bp)	Yes	No	10	0.098
VK210f	B (675–700 bp)	No	Yes	4	0.039
VK210g	B (675–700 bp)	No	No	13	0.127
VK210h	C (701–725 bp)	Yes	Yes	2	0.020
VK210i	C (701–725 bp)	Yes	No	5	0.049
VK210j	C (701–725 bp)	No	Yes	3	0.029
VK210k	C (701–725 bp)	No	No	6	0.059
VK210l	D (725–750 bp)	No	Yes	1	0.010
VK210m	E (750–775 bp)	Yes	No	4	0.039
VK210n	E (750–775 bp)	No	No	2	0.020
VK247A	A (650–675 bp)	No	ND	4	0.039
VK247B	B (675–700 bp)	No	ND	5	0.049
VK247C	C (701–725 bp)	No	ND	12	0.118
VK247D	D (725–750 bp)	No	ND	12	0.118
VK247E	E (750–775 bp)	No	ND	3	0.029

*ND* not done.

### Allelic diversity of *Pvmsp 1*

**Figure 4:**
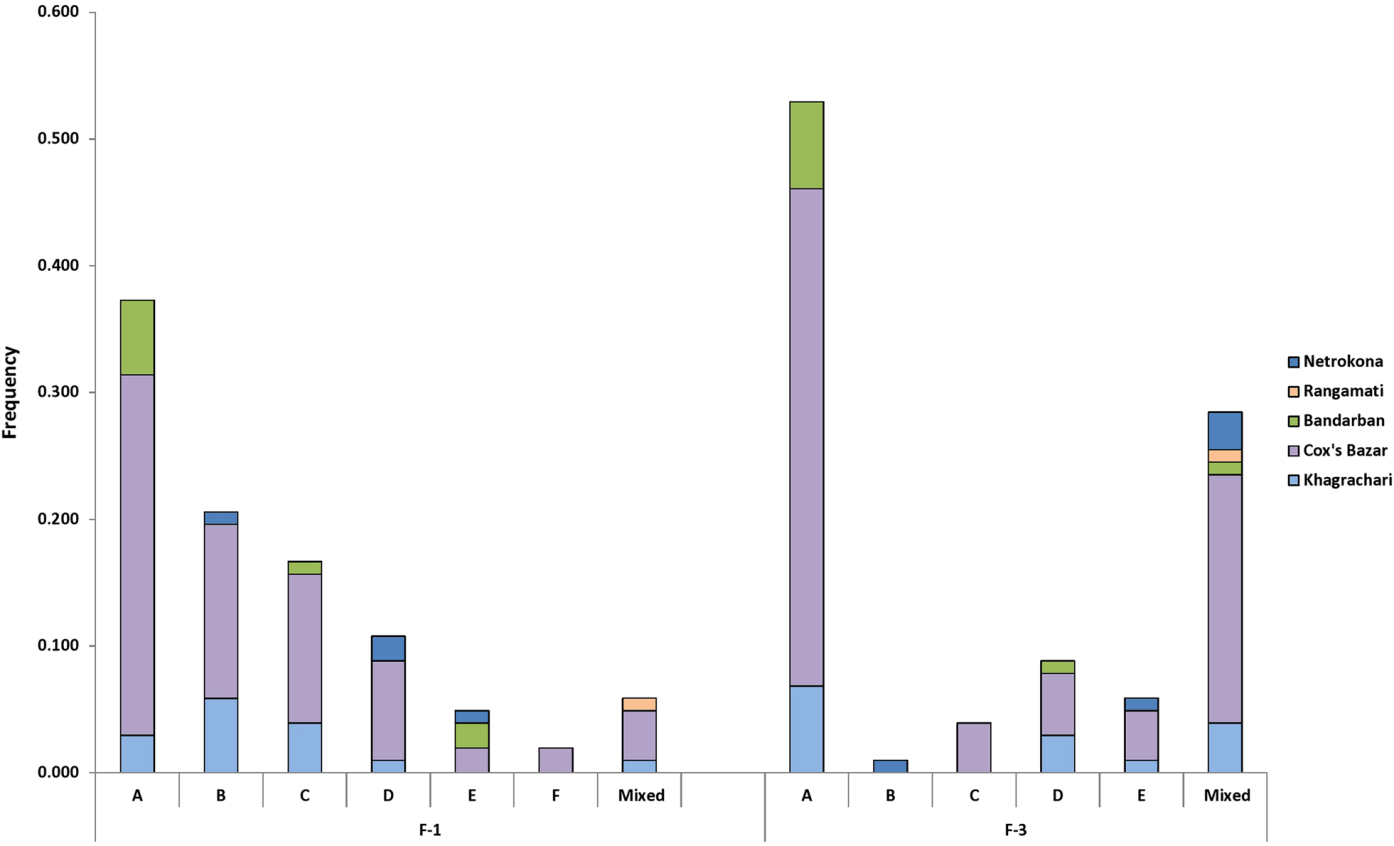
Size fragment distribution of *Pvmsp 1* F-1 and F-3 regions in five endemic areas.

A total of 56 distinct genotypes were observed in *Pvmsp 1* PCR. The geographical distribution for F1 and F3 region variants with their size variants (labelled alphabetically) is provided in Figure 4. For F1, six size variants (A–F), two (A and B) for F2 and three size variants (A–C) for F3 region were observed. For F1, variant A (350–374 bp) was the dominant variant (38% of the bands observed), variant B (1,200 bp) for F2 (57%) and variant A (250–274 bp) for F3 (62%) was the dominant allelic variant (Table 2; Figure 4). Increased genotype resolution was observed by RFLP for *Pvmsp 1* F2 fragment. In total, seven different *Alu I* patterns and five *Mnl I* patterns were observed (Table 2). Mixed patterns were also observed in both digestions where the sums of total band size exceeded the band size upon PCR. The highest frequency was found in Cox’s Bazar for both Ba7 (0.324) and Bm3 (0.333) in *Alu I* and *Mnl I* digestion, respectively (Table 2). Geographical distributions of all variants of these three regions (F-1, F-2 and F-3) are shown in Table 2 and Figure 4.

**Table 2:** Allele frequency of Pvmsp-1 F-2 region with geographical distribution

Allele	Size	n (frequency)				
		Khagrachari	Cox’s Bazar	Bandarban	Rangamati	Netrokona
Aa1	A	0 (0)	1 (0.010)	0 (0)	0 (0)	0 (0)
Aa2	A	0 (0)	2 (0.020)	0 (0)	0 (0)	0 (0)
Aa3	A	1 (0.010)	5 (0.049)	0 (0)	0 (0)	0 (0)
Aa4	A	0 (0)	0 (0)	1 (0.010)	0 (0)	0 (0)
Aa5	A	0 (0)	3 (0.029)	0 (0)	0 (0)	1 (0.010)
Aa6	A	2 (0.020)	3 (0.029)	1 (0.010)	0 (0)	0 (0)
Aa7	A	3 (0.029)	7 (0.069)	0 (0)	0 (0)	1 (0.010)
Ba3	B	0 (0)	1 (0.010)	0 (0)	0 (0)	0 (0)
Ba5	B	0 (0)	1 (0.010)	1 (0.010)	0 (0)	0 (0)
Ba7	B	3 (0.029)	33 (0.324)	3 (0.029)	0 (0)	1 (0.010)
Mixed		4 (0.039)	5 (0.049)	0 (0)	1 (0.010)	1 (0.010)
Not digested		1 (0.010)	5 (0.049)	3 (0.029)	0 (0)	0 (0)
Am1	A	0 (0)	2 (0.020)	0 (0)	0 (0)	0 (0)
Am2	A	0 (0)	0 (0)	1 (0.010)	0 (0)	0 (0)
Am3	A	4 (0.039)	15 (0.147)	1 (0.010)	0 (0)	2 (0.020)
Am4	A	1 (0.010)	3 (0.029)	0 (0)	0 (0)	0 (0)
Am5	A	0 (0)	1(0.010)	0 (0)	0 (0)	0 (0)
Bm2	B	0 (0)	1 (0.010)	0 (0)	0 (0)	0 (0)
Bm3	B	3 (0.029)	34 (0.333)	5 (0.049)	0 (0)	1 (0.010)
Bm4	B	0 (0)	1 (0.010)	1 (0.010)	0 (0)	0 (0)
Mixed		4 (0.039)	5 (0.049)	1 (0.010)	1 (0.010)	1 (0.010)
Not digested		2 (0.020)	4 (0.039)	0 (0)	0 (0)	0 (0)

### Allelic diversity of *Pvmsp 3α*

**Figure 5:**
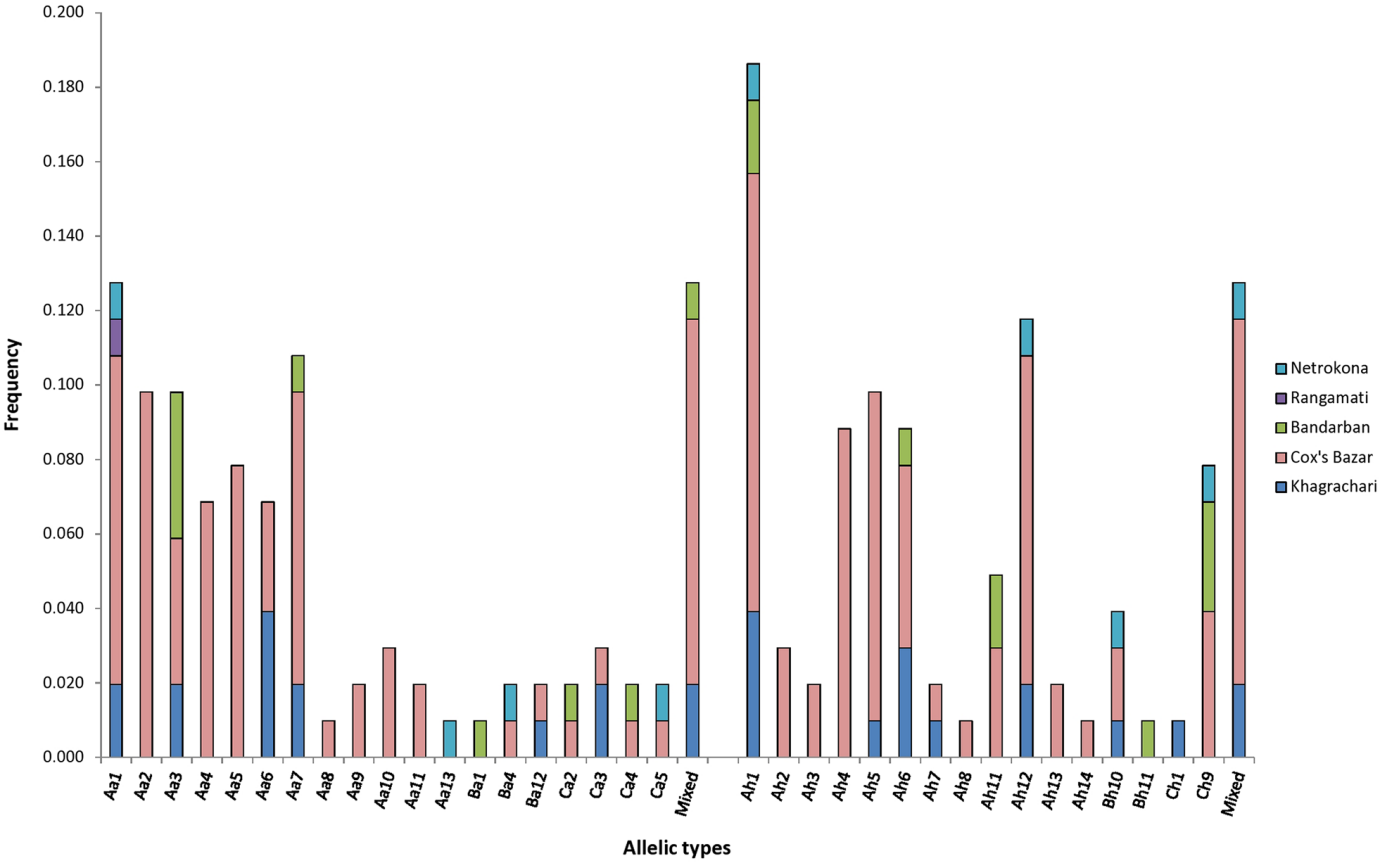
Geographical distribution of *Pvmsp* 3α marker using *Alu I* and *Hha I* restriction enzymes.

A total of 102 isolates were successfully amplified by nested PCR for *Pvmsp 3α,* which showed distinct size polymorphism with three allelic forms, labelled here as A, B and C. Seventy-five of these were A (1,900 bp), five of B (1,400 bp) and nine of C (1,100 bp). The remaining 13 isolates were mixed genotypes (Table 3). PCR–RFLP analysis by *Alu I* and *Hha I* restriction enzymes showed a greater genotype distinction among the isolates. *Alu I* digestion resulted in 19 different allelic variants for *Pvmsp 3α*, while *Hha I* digestion resulted in 16 distinguishable variants found from the study areas (Figure 5). A common clear restriction pattern of 1,000 bp fragment was observed in all samples in *Hha I* digestion (Figure 2). Thus, different patterns of smaller fragments (100–500 bp) were included for the genotyping analysis. Mixed infection was observed in 13 isolates with more than one PCR product of different sizes in a single sample or when the sum of the restriction fragments sizes exceeded the size of the PCR products.

**Table 3:** Size variant observations of *Pvmsp 3α*

	Area						
	Khagrachari	Cox’s Bazar	Bandarban	Rangamati	Netrokona	Total	Frequency
A (1,900 bp)	10	56	6	1	2	75	0.735
B (1,400 bp)	1	2	1	0	1	5	0.049
C (1,100 bp)	2	5	2	0	0	9	0.088
Mixed	2	10	0	0	1	13	0.127

The MOI is higher for *Pvmsp 1* (1.17) among these three markers. However, expected H_E_ is higher for *Pvcsp* but low in *Pvmsp 3α* (Table 4). Also, both MOI and H_E_ were calculated for each study year (Table 5) while low MOI and H_E_ were found only in 2012 for *Pvcsp* and *Pvmsp 3α*. Significant differences were observed for *Pvcsp* (*p* = 0.013), F1 and F2 variable regions of *Pvmsp 1* (*p* = 0.006 and 0.027, respectively) according to areas but not for others (*p* = 0.364 and 0.595, respectively for *Pvmsp 1* F3 region and *Pvmsp 3α*).

**Table 4:** Multiplicity of infection and expected heterozygosity (H_E_) of three different *P. vivax* polymorphic markers (based on PCR)

	Marker		
	*Pvcsp*	*Pvmsp 1*	*Pvmsp 3α*
Number of PCR positive samples (n)	102	102	102
Mean MOI	1.07	1.17	1.13
H_E_	0.777	0.689	0.437
${\text{P}} = \sum {\text{Pi}}^{ 2}$	0.230	0.318	0.567
Combined probability^a^		0.041	

*MOI* mean multiplicity of infection, *H*_*E*_ heterozygosity (expected).

^a^Calculated by multiplying the probabilities ‘P’ for all markers, which defines any of two independent clones share the same genotype.

**Table 5:** Multiplicity of infection and expected heterozygosity (H_E_) values according to years

Years	*Pvcsp*		*Pvmsp 1*		*Pvmsp 3α*	
	Mean MOI	H_E_	Mean MOI	H_E_	Mean MOI	H_E_
2009	1.14	0.81	1.21	0.72	1.14	0.66
2010	1.00	0.66	1.09	0.69	1.13	0.31
2012	1.00	0.00	1.33	0.72	1.00	0.00
2013	1.00	0.83	1.25	0.83	1.00	0.92

## Discussion

In this first report of genetic diversity of *P. vivax* in Bangladesh, a highly diverse *P. vivax* population was documented based on three markers *Pvcsp*, *Pvmsp 1*and *Pvmsp 3α* in malaria-endemic regions of the country.

Both VK210 and VK247 repeat types of *Pvcsp* were found in field isolates, where VK210 has higher prevalence. Previously, both the types were confirmed in a mosquito population from endemic areas of Bangladesh by CSP-ELISA where VK210 repeat types were also reported with higher prevalence [28, 29]. The prevalence of VK210 has been reported from other studies in Southeast Asia [9, 30, 31]. However, the VK247 repeat type is comparatively higher in Southeast Asia than other countries [18, 30–32]. Among the study areas, VK247 was observed highest in Netrokona (75%) followed by Bandarban (66.7%) of total *P. vivax* population in the respective areas, and none in Rangamati. The differences in frequency of VK247 type may be due to vector species distribution and their increased susceptibility to infection by VK247 repeat type and/or host immune pressure to certain *Pvcsp* repeat type [30, 32].

In this study, three different variable fragments (F1, F2 and F3) were analysed for *Pvmsp 1* marker and a total of 56 distinct variants were distinguished through size polymorphism and PCR–RFLP. This indicates extensive polymorphisms in the *Pvmsp 1* gene. In Thailand and India, less polymorphism has been reported [9, 30]. PCR–RFLP analysis showed high polymorphism in F2 fragments and out of a total of 102 PCR samples, seven different *Alu I* patterns and five different *Mnl I* patterns were observed, which is greater than other studies (Thailand, India, Pakistan) [9, 18, 30]. These variations indicate high genetic diversity in all the study areas and suggest that the *Pvmsp 1* gene is under selective pressure for the parasite’s survival and transmission [18].The *Pvmsp 3α* gene is a reliable molecular marker for genotyping study of *P. vivax*. Three different allelic variants were observed, of which size variant A (1,900 bp) was predominant as reported in other studies [30–33], but not in a study in Thailand [19]. Diverse RFLP patterns were observed for both *Alu I* and *Hha I* enzymes, with 19 and 16 distinct variants respectively, which is fewer than reported elsewhere [31, 32], but almost the same as another study from India [30]. While the findings are not directly comparable, with a dissimilar sampling strategy in use in this study, these values are strikingly higher in respect of the low endemicity of vivax malaria in certain regions, such as Thailand. Biological features of *P. vivax,* such as earlier gametocytogenesis, ie., production of gametocytes in the presymptomatic period before the drug treatment is initiated, and relapse could be the reason behind this extensive polymorphism. Earlier gametocytogenesis and relapses might allow for more efficient transmission to the vector mosquitoes [15].

In this study, extensive genetic diversity of all three markers in *P. vivax* populations was observed in Bangladesh. Diverse anopheline fauna and their susceptibility to infections by different parasite types [28, 34] can be vital reasons for this diversity. Most of the study areas are populated with different ethnic groups and there may be the presence of different host immune responses to the parasite, which can support this diversity. Also, migration of people from one country to another may carry different parasite variants that increase diversity to the gene pool [32], which is common on the Bangladesh-Myanmar-India border areas.

## Conclusion

High genetic diversity based on *Pvcsp, Pvmsp1* and *Pvmsp3α* for *P. vivax* in clinical isolates was observed in Bangladesh. Establishment of genotyping methods for these three polymorphic markers and the knowledge from this study will provide valuable support for future genotyping study of recurrent infection, which will help in drug efficacy and drug resistance observation.
